# A post-lockdown online cross-sectional survey on knowledge, attitude and anxiety levels of Nigerian youths towards COVID-19 disease

**DOI:** 10.11604/pamj.2022.42.178.34451

**Published:** 2022-07-06

**Authors:** Batholomew Chibuike James, Chinazaekpere Mary Aroh, Stephen Sunday Ede, Felix Emeka Anyiam, Michael Ikechuwu Uhuo, Kanokwan Chullapant, Esthinsheen Osirim, Mathias Nwojiji

**Affiliations:** 1 Public Health Program, Graduate School, Angeles University Foundation, Angeles City, Pampanga, Philippines,; 2 Royal Berkshire National Health Service Foundation Trust, Reading, United Kingdom,; 3 Department of Medical Rehabilitation, College of Medicine, University of Nigeria, Enugu, Nigeria,; 4 Centre for Health and Development, University of Port Harcourt (UNIPORT), River State, Port Harcourt, Nigeria,; 5 Endocrinology and Metabolism Unit, Prince Songkhla University Hat-Yai, Songkhla Province, Thailand,; 6 Department of Physiology, Faculty of Basic Medical Sciences, Redeemer's University Ede, Osun State, Nigeria,; 7 Department of Soil Science and Environmental Management, Ebonyi State University, Ebonyi State, Nigeria

**Keywords:** COVID-19, knowledge, attitudes, anxiety level, Nigerian youths

## Abstract

**Introduction:**

COVID-19, also known as Coronavirus disease, was detected in Wuhan, Hubei, China in December 2019. Since then, the virus has been designated a global pandemic, affecting all nations. Nigeria as a whole has recorded 255,937 cases of COVID-19. Studies on COVID-19 anxiety level, knowledge, and attitude have not been focused on youths after the lockdown. This research explored COVID-19-related knowledge, attitudes, and anxiety levels among Nigerian youths after the lifted pandemic lockdown.

**Methods:**

after the COVID-19 pandemic lockdown, an online descriptive cross-sectional survey was performed among 818 young people aged 18 to 35 in South-Eastern Nigeria. The Statistical Package for Social Science (SPSS) version 25 technique was used to analyze the descriptive statistics. The Chi-square test, bivariate, and multivariate binary logistic regression were used to measure the associations. A p-value of 0.05 was statistically significant at a 95% confidence level.

**Results:**

the majority of respondents 723 (88.39%) demonstrate a high degree of COVID-19 knowledge. Just a few respondents (0.49%) expressed a negative understanding of the virus. 556 (67.97%) had a positive attitude towards the virus. More than half of the respondents had a low anxiety level, 471 (57.58%) and 108 (13.20%) had a high level. Educational level, place of residence, and family income showed a statistically significant relationship with their anxiety levels (p = 0.001, 0.002, 0.01, respectively).

**Conclusion:**

the infection, transmission, and symptoms of COVID-19 were well-understood by young people after the pandemic, who also displayed optimistic attitudes and low levels of fear. Higher levels of education, family income, and dwelling location were connected with COVID-19 knowledge and lower anxiety levels. This knowledge will assist health professionals in fighting current and future related outbreaks.

## Introduction

In December 2019, the Coronavirus Disease known as COVID-19 was identified in the Chinese province of Hubei, Wuhan. The disease is associated with a novel coronavirus, the seventh known virus to infect humans [1,2]. The virus belongs to the family of respiratory viruses, which bring about the common cold, Middle East Respiratory Syndrome (MERS), and severe acute respiratory syndrome (SARS) [3]. The virus came to be known as severe acute respiratory syndrome coronavirus 2 (SARS-CoV-2) [4] in 2019. Since then, the virus has been labelled a worldwide pandemic afflicting all countries and connected with direct morbidity and mortality burdens [5]. However, a WHO study indicated that the death rate was between 3 and 4% [6]. Even though Baud *et al*. indicate that death rates are underestimated [7]. This has necessitated the implementation of several disease control and preventive measures with significant social and economic repercussions globally [4]. As of May 20^th^, 2022 Nigeria as a whole had registered 255,937 COVID-19 cases, 3,143 fatalities, and 249,996 discharges; nevertheless, the South-Eastern States of Nigeria were not among the top ten States in Nigeria with the highest number of COVID-19 cases but have recorded 12,580 cases and 172 deaths [8]. In a bid to control the spread of the disease, numerous nations enforced countrywide lockdowns per World Health Organization (WHO) recommendations to help avoid the collapse of global health systems[9].

On March 30^th^, 2020 international, national, and interstate transport connections, marketplaces, offices, and commercial transactions, schools (primary and secondary), sports, religion, and all other relevant social gatherings were restricted as containment measures to limit the spread of the virus [10]. However, Nigeria has not been an exception to the effects of this pandemic, even though the ban was progressively lifted in three stages from the 4^th^ of May 2020, to the 10^th^ of May 2021 [6,9]. In addition to the virus's association with morbidity and death, there have been significant mental health consequences. Isolation and quarantine as a result of the development and spread of the new coronavirus were accompanied by the devastation on a worldwide scale, including anxiety, nervousness, panic, depression, and post-traumatic stress symptoms among the general population [5,11]. Since the inception of the pandemic, the incidence of anxiety disorders has risen compared to the level before the pandemic [12-14]. This trend has also been documented in Nigeria. It may be attributable to several variables, such as the unreliable flow of information about the pandemic, social distancing efforts, loss of jobs or money, the recurrent incidence of robberies, and reports of food shortages and hunger [15,16]. It is assumed that the COVID-19 crisis is a long-term process. Nevertheless, the success of disease-related literacy in preventing infectious illnesses and pandemics has been well reported in the research and literature [17]. Appropriate health promotion will help increase knowledge, resulting in a shift in undesirable behaviors and attitudes. It will also shape the reactions and practices of individuals whilst controlling spread of the disease in the face of a pandemic [18]. Due to Nigeria's relatively youthful population, with a median age of 18.4 years, youths may serve as change agents by distributing vital knowledge [19,20]. This research examined the relationship between COVID-19-related anxiety, attitudes, and knowledge among young Nigerians after the lifted pandemic lockdown. Addressing knowledge and attitude gaps about the disease is essential and would provide more understanding, assisting in the control and management of the present pandemic and maybe prevent future outbreaks. It will also give insight into how the public's anxiety levels have shifted after the restrictions were lifted. Most of this information may serve as an essential baseline for public health stakeholders and authorities, helping to build anxiety-free educational and health programs for the communities

## Methods

**Study design and setting:** this was an online correlation and cross-sectional research, sampled among young people living in South-Eastern Nigeria mostly dominated by Igbo tribe. The South-Eastern States were chosen to enable the researchers to give clarity to the outcomes that are to be investigated and to facilitate comparison between future studies. These States include Anambra, Enugu, Imo, Aba, and Ebonyi. The area is home to over 22 million people and approximately 10% of Nigeria total population [21]. After the pandemic lockdown was lifted, the researchers recruited study participants and gathered data using an online platform between June and September 2021. To prevent the transmission of COVID-19 between participants and researchers, a web-based questionnaire was used.

**Study participants:** the research population consisted of Nigerian young adults between the ages of 18 and 35 who resided in the study region. Inclusion criteria for study participants were residency in the Southeast and understanding of the English-language questionnaire. Those who did not give their consent and those who have not lived in the study region for the past three months prior to the data collection were excluded from this study.

**Sample size estimation:** Taro Yamane formula was used in calculating the sample size to be 400 participants at a confidence level of 95% and 5% margin of error for every question [22]. The formula States that:


$$n=\frac{N}{1+N(e^{2})}$$

Where: n = sample size, N = population size, e = marginal error The sample size was raised by 100% to account for non-response and attrition, reduce sample variability and improve generalization. To gather the required number of study participants, a method of convenience sampling was used. This is a non-probability sampling technique that selects participants depending on their availability [23]. Only questionnaires with all sections completed by respondents were used for data analysis, and after data collection, 818 respondents were selected for data analysis.

**Study instrument:** the survey tools were divided into four parts. Age, sex, marital status, education, employment, median household income, and ethnicity were collected in the first part. In the second part, participants were assessed on their awareness of the COVID-19 pandemic through several questions as adapted in prior research by Lin *et al*. [14]. The total knowledge score for each participant was determined by awarding a score of 0 for each question with an incorrect answer and 1 for each question with a right response, and then adding the scores. The results were then converted to a percentage (out of a total of 14 points), and the participant´s knowledge was divided into three categories of expertise based on arbitrary cutoff criteria: low (0 percent to 30 percent score), fair (31 percent to 60 percent score), and good (61- 100 percent). The third part questioned respondents' attitudes on the COVID-19 pandemic. Ten questions were modified from published research by Zhong *et al*. [24] were used to assess attitudes. Included in the evaluation questions were statements with three Likert-scale replies (“Yes”, “Not sure”, and “No”). Each “Yes” answer was allotted 2 points, each “Not sure” response was awarded 1 point, and each “No” response was assigned 0 points. The attitude scores were tallied and translated to percentages (out of 20 points), and the participants were grouped into three groups: poor (0 percent to 30 percent), fair (31 percent to 60 percent), and good (61 percent to 100 percent). The fourth part of the questionnaire measured anxiety due to the COVID-19 pandemic with 15 Likert-scale items previously used questions in a survey by Lin *et al*. [18]. Questions were designed using a four-point Likert scale depending on the degree of agree ability: “Very much so,” “Somewhat so,” “Somewhat,” and “Not at all,” with commensurate points of 3, 2, 1, and 0 allocated. Reverse coding was used for negative statements. The anxiety ratings were then converted to percentages (out of 45 potential points) and the participants´ responses were divided into three categories of anxiety: low (0-30 percent), moderate (31-60 percent), and high (61 percent - 100 percent). Two medical researchers and data scientists evaluated the questionnaire's construct validity, and their suggestions were adopted. The survey tools were also pilot-tested on fifteen adults selected through the face-to-face survey method, but the results were excluded from the study. It's reliability was measured using Cronbach's alpha. The result was 0.85, confirming its reliability.

**Study procedure:** using an online Google form, the modified assessment tool was developed. It was made accessible to every eligible Nigerian young adult from five South-Eastern States who met the study's eligibility requirements. Invitations to participate in the research were disseminated on social networking sites using a “one-time-only link” to the online survey to prevent duplicated responses. After clicking on this “one time only” link to the questionnaire, participants were immediately sent to the survey's entry page, which included details on the study's objectives, informed consent, participation criteria, data privacy, and possible risks and benefits of the survey. The completion time for the survey in one sitting was between 15 and 25 minutes. If the questionnaire was not completed in a single sitting, the “one time only” link was automatically disabled. Conveniently, respondents may access and finish the survey on a smartphone or computer.

**Ethical consideration:** the health research ethics committee of the University of Nigeria Teaching Hospital, Ituku/Ozalla, Enugu, Nigeria, granted ethical permission for this study. (Authorization number: NHREC/05/01/2008B-FWA00002458-1RB00002323). The participants consented to have their replies anonymized and collated for publishing and research purposes. Only those who agreed to participate were directed to the survey site automatically.

**Data analysis:** a Microsoft Excel spreadsheet was used for data editing, sorting, and coding. Version 25 of the Statistical Package for Social Science (SPSS) software was used for data analysis. This procedure was completed on a password-protected computer to guarantee the greatest discretion. The data analysis comprised descriptive statistics (frequency, percentage, mean, standard deviation) that illustrated the distribution of research participants with the outcomes of interest. We conducted a Chi-square test to establish the connection between sociodemographic characteristics and anxiety levels. We next utilized bivariate and multivariate logistic regression analysis to investigate the degree to which knowledge and attitude about the COVID-19 pandemic were linked with COVID-19-related anxiety. A p-value of less than 0.05 was deemed statistically significant at the 95% confidence level.

## Results

**Respondents' sociodemographic characteristics:** the majority of the 818 respondents to this online survey were male (56.1%), between the ages of 25 and 29 (49.14%), lived in an urban region (67.52%), had a Diploma or BSc as their highest degree, and had an average household income of >90,000 Nigerian Naira (31.7%). Moreover, most of the respondents were employed (44.50%) and unmarried (83.62%); since this study was conducted in a South-Eastern region of Nigeria, a higher percentage of respondents understands Igbo (79.78%), while Hausa (1.96%) and Yoruba (38 or 4.66%) were the uncommon spoken languages in the region (Table 1).

**Table 1:** sociodemographic characteristics of respondents

Characteristics	Frequency (n)	
Age (years)		Percentage (%)
18-24	178	21.76
25-29	402	49.14
30-35	238	
**Gender**		29.10
Male	459	56.11
Female	359	
**Marital status**		43.89
Single	684	83.62
Married	134	
**Educational level**		16.38
Primary	6	0.73
Secondary	24	2.93
Diploma/BSc	653	79.83
MSc/PhD	135	16.50
**Occupational status**		
Employed	364	44.50
Self-employed	208	25.43
Student/ Not working	246	
**Average household income in Nigerian Naira**		30.07
<15,000	152	18.58
15,001-30,000	167	20.42
30,001-60,000	138	16.87
60,001-90,000	106	12.96
> 90,000	255	
**Residential location**		31.17
Urban	553	67.60
Semi-urban	196	23.96
Rural	69	
**State of residence**		8.44
Enugu	261	31.91
Abia	103	12.59
Ebonyi	118	14.43
Imo	138	16.87
Anambra	198	24.20

**Knowledge about the COVID-19 infection after the pandemic lockdown:** knowledge of COVID-19-related illnesses (Table 2) illustrates that every respondent had heard about COVID-19, with 652 (79.71%) hearing about it through the internet or social media. Most of them answered correctly when asked whether COVID-19 is the same as the flu virus 545 (66.63%), what causes COVID-19 744 (90.95%), if eating or touching wild animals might result in COVID-19 infection 530 (64.79%), if it is feasible for a COVID-19-positive individual to exhibit no symptoms 663 (81.05%), who can be infected with COVID-19 802 (98.04%), and if handwashing is vital for preventing COVID-19 infection. When asked what disease(s) they believe are comparable to COVID-19, the majority answered incorrectly, with 258 (31.54% selecting malaria and 228 (27.87%) selecting SARS. Also, the majority of respondents, 691 (84.47%,) believed that the incubation time for COVID-19 is 1-4 days, whereas 40 (4.89%) percent believe it is 2-21 days. In addition, the table revealed that the majority of respondents were able to identify COVID-19's symptoms, its mode of transmission, and preventative measures. In general, 723 (88.39%) of respondents demonstrated good knowledge of COVID-19 infection, transmission, and prevention, while 91 (11.12%) demonstrated fair knowledge and just 4 (0.49%) demonstrated poor knowledge of the disease after the lockdown was lifted (Table 2).

**Table 2:** knowledge about COVID-19 transmission and symptoms

Knowledge	Frequency (n)	Percentage (%)	Knowledge	Frequency (n)	Percentage (%)
Have heard of COVID-19	818	100	list symptoms of COVID-19		
**Source of information about COVID-19 µ**			High fever	685	83. 74
Internet/social media	652	79.71	Fatigue	228	27.87
TV	76	9.29	Dry cough	192	23.47
**Government enlightenment campaign**	144	17.60	Muscle pain	124	15.16
Friends and family	21	2.57	Runny nose	17	2.08
Newspaper	12	1.47	Bleeding	13	1.59
Church and outreach	5	0.6	Breathing difficulty	308	37.65
**COVID-19 virus is the same as the Flu virus?**			**How does the virus spreads**		
Yes	175	21.39	Air droplets (from patient sneezing/coughing)	750	91.69
No	545	66.63	Close contact with people who have the virus	243	29.71
I don't know	98	11.98	**Contact with contaminated surfaces**	22	2.69
**Participant's response when asked what causes COVID-19**			Mosquitoes/flies	1	0.12
Virus	744	90.95	I don't know	10	1.22
Bacteria	23	2.81	**What can kill the COVID-19 virusµ**		
Fungi	3	0.37	Alcohol-based sanitizers	380	46.45
I don't know	48	5.87	Soap/detergents	52	6.36
**Eating or contacting wild animals results in COVID-19 infection?**			Clean surfaces with diluted chlorine	438	53.55
Yes	157	19.19	**The length of time hand washing takes to kill the virus**		
No	530	64.79	Less than 20s	130	15.89
I don't know	131	16.01	The 20s to 1 min	646	78.97
**Which disease(s) do you think are similar to COVID-19?µ**			I don't know	42	5.13
SARS	258	31.54	**Hand washing is important in the prevention of the virus**		
Malaria	228	27.87	Yes	810	99.02
Ebola	191	23.35	No	2	0.24
Typhoid	45	5.50	Maybe	6	0.73
HIV/AIDS	24	2.93	**Who can be infected with COVID-19**		
None of the above	72	8.80	Anyone can be infected	802	98.O4
**COVID-19 positive person could show no symptoms?**			Young adults only	8	0.98
Yes	663	81.05	White people only	6	0.73
No	127	15.53	Older people only	2	0.24
I don't know	28	3.42			
**How long does it take for the COVID-19 Symptoms to show-up**			**Participants overall knowledge of COVID-19**		
Less than 7 days	52	6.36	Poor (0%-30% **score**)	4	0.49
1-4 days	691	84.47	Fair (31%- 60% **score**)	91	11.12
2-21 days	40	4.89	Good (61%-100% **score**)	723	88.39
I don't know	35	4.28			

µ This is a ''Yes'' and a ''No'' option questions and participants are allowed to tick more than one option (multiple responses apply), with the sample size being the denominator

**Attitudes towards COVID-19 infection and transmission after the pandemic lockdown:** in general, most respondents, 556 (67.97%), had positive attitudes about COVID-19 transmission and symptoms. Specifically, the majority of respondents correctly responded that regular hand washing might reduce the risk of coronavirus infection 768 (93.89%), that they would isolate if exhibiting symptoms such as fever and cough 603 (73.72%), they also agreed that social distance is necessary to stop the spread 751(91.81%). They are willing to read and share accurate information about COVID-19 770 (94.13%), high a number of the respondents trust the social media coverage of the COVID-19 pandemic 770 (94.13%) (Table 3). In contrast, just 100 (12.22%) of them acknowledged that travelling across/within the nation during pandemic periods is unsafe, while only 327 (39.98%) stated they would get the COVID-19 vaccine. Patients with COVID-19 who have been certified cured should be permitted to remain in the community, was acknowledged by only 157 (19.19%) of the respondents (Table 3).

**Table 3:** attitude towards COVID-19 transmission and symptoms

Characteristics	''Yes'' (2 points)	Not sure (1 point)	''No'' (0 points)
Think washing hands frequently can lower the risk of coronavirus infection	768 (93.89)	14 (1.71)	36 (4.40)
How likely to quarantine/isolate if you have fever and cough	603 (73.72)	101 (12.35)	114 (13.94)
Think social distance is essential to stop the spread	751 (91.81)	24 (2.93)	43 (5.26)
Think traveling across/within the country is safe during these times	125 (15.28)	593 (72.49)	100 (12.22)
Will you accept the COVID-19 vaccine	327 (39.98)	291 (35.57)	200 (24.45)
Patients with COVID-19 who are declared cured, should not be allowed to stay within the community at this time	170 (20.78)	491 (60.02)	157 (19.19)
Willing to read and share with others the right information about COVID-19	770 (94.13)	12 (1.47)	36 (4.40)
Believe COVID-19 in Nigeria is real	593 (72.49)	118 (14.43)	107 (13.08)
Government response towards controlling COVID-19 satisfactory	94 (11.49)	629 (76.89)	95 (11.61)
Media/social media coverage of the COVID-19 pandemic satisfactory?	421 (51.47)	274 (33.50)	123 (15.04)
**Overall attitude towards COVID-19**	**Frequency**	**Percent**	
Poor (0%-30% score)	9	2.32	
Fair (31%-60% score)	243	29.71	
Good (61%-100% score)	556	67.97	

**Anxiety related to COVID-19 infection after the pandemic lockdown:** as shown in (Table 4), the respondents expressed an overall low level of anxiety related to the COVID-19 pandemic (57.58%). Most respondents indicated that they are very calm (58.58%), a comfortable (37.53%) about the pandemic. And, the majority stated “Not at all” when they were tense (63.33%), upset (57.33%), worrying over possible misfortunes (44.99%), frightened (63.33%), nervous (57.95%), worried (50.61%), confused (66.75%), and (49.02%) having disturbing thoughts (Table 4).

**Table 4:** anxiety related to COVID-19 pandemic

Characteristics	Very much so	Moderately so	Somewhat	Not at all
	(3 points)	(2 points)	(1 point)	(0 points)
I feel calm*	471 (58.58)	247 (30.20)	73 (8.92)	27 (3.30)
I am tense	38 (4.65)	121 (14.79)	141 (17.24)	518 (63.33)
I feel upset	83 (10.15)	91 (11.12)	175 (21.39)	469 (57.33)
I am presently worrying over possible misfortunes	117 (14.30)	120 (14.67)	213 (26.04)	368 (44.99)
I feel satisfied*	244 (29.83)	278 (33.99)	156 (19.07)	140 (17.11)
I feel frightened	65 (7.95)	92 (11.25)	143 (17.48)	518 (63.33)
I feel comfortable*	307 (37.53)	258 (31.54)	138 (16.87)	115 (14.06)
I feel nervous	63 (7.70)	108 (13.20)	173 (21.15)	474 (57.95)
I am worried	104 (12.71)	105 (12.84)	195 (23.84)	414 (50.61)
I feel confused	67 (8.19)	81 (9.90)	124 (15.16)	546 (66.75)
I wish I could be as happy as others seem to be	232 (28.36)	122 (14.91)	124 (15.16)	340 (41.56)
I am calm, cool, and collected*	489 (59.78)	214 (26.16)	83 (10.15)	32 (3.91)
I have disturbing thoughts	123 (15.04)	121 (14.79)	173 (21.15)	401 (49.02)
I make decisions easily*	187 (22.86)	331 (40.46)	176 (21.52)	124 (15.16)
I get in a state of tension or turmoil as I think over my recent concerns and interest	128 (15.65)	195 (23.84)	219 (26.77)	276 (33.74)
**Overall anxiety level**	**Frequency (n)**		**Percentage (%)**	
Low (0-30% score)	471		57.58	
Moderate (30- 60% score)	239		29.22	
High (60- 100% score)	108		13.20	

Key: * responses scored in reverse

**Anxiety levels are linked to sociodemographic factors of the participants:** the study shows higher odds for moderate to high anxiety among participants with a secondary level of education or less (cOR=5.29, 95% CI: 2.26-12.37) or Diploma/BSc (cOR=2.12;95% CI: 2.26-12.37) compared to those with MSc/PhD (p>0.001). Also, higher odds for moderate to high anxiety among participants were observed among those residing in the rural or semi-urban region (cOR=1.49; 95% CI: 1.11-2.03) compared to those living in the urban area (p>0.01). Increasing anxiety was observed among those with a lower average household income compared to those with a high average monthly income (Table 5).

**Table 5:** association between sociodemographic characteristics and anxiety level among survey participants after the pandemic lockdown

Anxiety levels			
Variables	Low (n=471)	Moderate/high (n=347)	P-value*
**Age (years)**			
18-24	102 (57.30)	76 (42.70)	0.177
25-29	243 (60.45)	159 (39.55)	
30-35	126 (52.94)	112 (47.06)	
**Gender**			
Male	260 (56.64)	199 (43.36)	0.54
Female	211 (58.77)	148 (41.23)	
**Educational level**			
≤Secondary	10 (33.33)	20 (66.67)	0.001*
Diploma/BSc	363 (55.59)	290 (44.41)	
MSc/PhD	98 (72.59)	37 (27.41)	
**Residential area**			
Rural/semi-urban	135 (50.94)	130 (49.06)	0.01*
Urban	336 (60.76)	217 (39.24)	
**Average household income**			
<15000	80 (52.63)	72 (47.37)	0.002*
15000-30000	81 (48.50)	86 (51.50)	
30000-60000	77 (55.80)	61 (44.20)	
>60000	233 (64.54)	128 (35.46)	

* Statistically significant (p<0.05); Based on chi-square test of association

**Association between knowledge, attitude, and anxiety levels:** a further regression analysis presented in (Table 6) showed when a non-adjusted (crude) aggregate model comprising the explanatory variable knowledge was associated with anxiety levels, there were higher odds for moderate to high anxiety among participants with a secondary level of education or less (cOR=5.29, 95% CI: 2.26-12.37) or Diploma/BSc (cOR=2.12;95% CI: 2.26-12.37) compared to those with MSc/PhD. Additionally, the study shows higher odds for moderate to high anxiety among participants with fair/poor knowledge than those with good knowledge (cOR=2.32, 95% CI: 1.38-3.91). This study observed no statistically significant association existed between attitudes and anxiety (p>0.05). The odds of participants experiencing higher levels of anxiety only increased by 1.02 (cOR=1.02, 95% CI: 0.75-1.37) when attitude was kept constant (Table 6).

**Table 6:** logistic regression analyses on the association between knowledge, attitude, and anxiety levels after the lockdown

Variables	Anxiety levels		Model 1^a^	Model 2^b^
	Moderate/high (n=347)	Low (n=471)		
**Educational level**				
≤Secondary	20 (66.67)	10 (33.33)	5.29*	0.16
			(2.26-12.37)	(0.069-0.39)
Diploma/BSc	290 (44.41)	363 (55.59)	2.12*	0.41
			(1.41-3.18)	(0.27-0.63)
MSc/PhDR	37 (27.41)	98 (72.59)	-	-
**Residential area**				
Urban	217 (39.24)	336 (60.76)	-	-
Rural/semi-urban	130 (49.06)	135 (50.94)	1.49*	1.51*
			(1.11-2.03)	(1.12-2.03)
**Average household income**				
<15000	72 (47.37)	80 (52.63)	1.64 *	0.58*
			(1.12-2.41)	(0.39-0.87)
15000-30000	86 (51.50)	81 (48.50)	1.93*	0.49*
			(1.33-2.80)	(0.34-7.24)
30000-60000	61 (44.20)	77 (55.80)	1.44	0.65*
			(0.97-2.15)	(0.43-0.98)
>60000R	128 (35.46)	233 (64.54)	-	-
**Knowledge**				
Good	307 (40.77)	446 (59.23)	-	-
Fair/poor	40 (61.54)	25 (38.46)	2.32*	2.42*
			(1.38-3.91)	(1.43-4.08)
**Attitude**				
Good	235 (42.27)	321 (57.73)	-	-
Fair/poor	112 (42.75)	150 (57.25)	1.02	1.03
			(0.75-1.37)	(0.77-1.39)

Statistically significant (p<0.05); R=reference; a) Model 1: crude OR (95% CI); b) Model 2: adjusted OR (95% CI), adjusted for age and sex

## Discussion

The novel coronavirus disease is one that has brought about a significant impact on the different areas of life and well-being. This study assessed the knowledge, attitudes, and anxiety level of youths (18-35 years old) in South-Eastern Nigeria following the easing of the COVID-19 lockdown and restrictions. The study reveal that most respondents demonstrated good knowledge and attitude after the COVID-19 lockdown was lifted. In addition, socio-demographic factors and respondents´ knowledge of the pandemic was associated with anxiety level. In contrast, there was no association between anxiety level and attitudes of the respondents towards the pandemic. This is further discussed in detail in the sections that followed.

**Knowledge of COVID-19 infection, transmission, and symptoms after the pandemic lockdown:** the study revealed that a large proportion (88.39%) of the study participants have good level of knowledge of COVID-19 infection. The transmission routes, incubation period, symptoms, and preventive measures were well recognized by the respondents. Given that majority of the respondents had attained a tertiary level of education, the high level of knowledge on COVID-19 found in this study was predictable. The findings are similar to a study conducted in Nigeria among adult residents during the ease of the lockdown, which also reported that a very large proportion (98.8%) of the respondents had a good knowledge of the disease. However, when compared to other published studies carried out in the early stages of the pandemic and before the ease of the restrictions [25-27] we found that the knowledge levels were significantly improved post lockdown. The internet/social media was also the main source of information for this group. This is not surprising as the internet and social media have evolved into a tool rampantly utilized by young people not just for social interaction but one that is highly accessible and with scalable publishing methods [28]. This depicts the effectiveness of health promotional campaigns launched on various media platforms by the Federal Ministry of Health (FMOH) and the National Centre for Disease Control (NCDC) since the start of the pandemic to avert its spread. The finding is an indicator that the internet can fairly be relied upon for advocacy and informative purposes in times of crises or disease outbreaks and is also in accordance with other results that reported the use of social media as the main tool for COVID-19 information [24,29-31]. Nonetheless, the cut-off points for a “good” level of knowledge in our study were arbitrarily determined strictly based on the spread of the grades, and the awareness marks evaluated in our research may not directly correlate to the thought process that served as the drivers of having to engage in COVID-19 prevention and treatment actions [32]. In addition, several questions, such as the source of information on COVID-19, did not assess knowledge directly. In this research, construct validity of understanding measuring questions was a primary concern.

**Attitude towards COVID-19 infection, transmission, and symptoms after the pandemic lockdown:** there was a good attitude (67.97%), related to COVID-19 infection transmission, symptoms, and prevention after the lift of the lockdown. However, in promoting positive attitudes and health behaviors among individuals, the role of knowledge cannot be over-emphasized [10]. The respondents had confidence that by adopting preventive measures such as hand washing, social distancing, and isolation, the risk for infection spread is lowered. This indicates that most of the respondents still pay attention to infection prevention and control measures even after restrictions have been eased. This result was different from the early stage of the COVI-19 pandemic lockdown. A survey conducted in northern Nigeria in 2020, shows that 17.8% of the respondents expressed positive attitudes and another study in South-Eastern Nigeria show an average report among the participant [26,33]. However, similar reports were recorded from a Malaysian and Chinese study during the later stage of the COVID-19 pandemic in which the respondents agreed that COVID-19 would finally be controlled successfully [34,35]. Meanwhile, one-third of the respondents expressed fair and poor attitudes (29.71%) and (2.32%) respectively. The respondents´ responses could be reflected in a previous study on SARS regarding their uncertainty about the safety of travelling across/within the nation during pandemic periods and stigmatization towards previous positive patients in the community [36]. And also, the unwillingness to accept the vaccine and their dissatisfaction with governments´ response in controlling the pandemic [37,38]. This shows that misconceptions about the COVID-19 infection still exist and people do not pay enough attention to the vaccine. There is therefore the need for the government and media to scale up the popularization of vaccine-related COVID-19 knowledge. Also, in line with the recommendations of previous literature, it is further imperative that community-based health campaigns are instituted to sustain positive attitudes and erode misconceptions [38]. They were problems with the questions used to measure attitudes. Attitude is how a person feels about a certain action. Some of the attitude measurement questions, like whether or not people are willing to take the COVID-19 vaccine or whether or not people who have recovered from COVID-19 should be judged, could be seen as attitudes toward COVID-19 prevention efforts. However, other questions, like how effective hand hygiene is at reducing the risk of infection and whether or not COVID-19 is real in Nigeria, don't seem to accurately reflect this concept. When trying to figure out what our findings about attitudes mean, care should be taken about the construct validity. As has been done in other places, future studies should think about asking questions about practices, behaviors, and beliefs [32].

**Respondents' anxiety levels towards the COVID-19 disease after the lockdown:** this current study reveals a lower level of anxiety 471 (57.58%) among the study participant after the lift of the lockdown. In contrast, previous studies during the lockdown show high level of anxiety in Nigeria, and China populations [12,39]. Perhaps this may be due to the inconsistency of information in the first phases of outbreak and restriction. However, some studies during the early pandemic and lockdown were consistent with the current findings. Studies in Oman and Australia population showed a low level of anxiety among the population during the early stage of the lockdown [40,41] and another study conducted in the South-Eastern part of Nigeria also recorded a low level of anxiety among the young population in that region during the lockdown [42]. This could be related to accurate information dissemination and government support systems among the population. However, significant limits occurred in our measuring of anxiety. We adopted an ad hoc tool seen in prior reports in China [18] rather than the basic tool for the assessment of anxiety disorders, such as the generalized anxiety disorder 7 (GAD-7) checklist, which had developed sensitivity and specificity at specific cut-off scores and would have been used and validated in so many prior studies [43]. The accuracy of our assessment was therefore compromised.

**Anxiety levels linked to socio-demographic characteristics of the participants:** the respondents´ residential area, family income, and educational qualification were statistically associated with anxiety level related to the COVID-19 pandemic, as those with secondary education or less, and those with Diploma/BSc have high and moderate level of anxiety, compared to respondents with MSc/PhD who have low anxiety level. These findings were consistent with a survey in 2020 among Australia population, where those with PhD have low anxiety level [41]. Another study also mentioned that educational level plays a role in a person stress level [44]. This research demonstrates that academic experience positions young people to have greater access to reliable information [45]. Which concludes that, people with a higher level of education exhibited more informed responses than those with a lower level of education. Health education dissemination programs should target those with lesser academic qualification. Household income was further associated with respondent anxiety level. This finding was similar to Chinese population, which observed an impact of household income is associated to anxiety [18]. Also a study in the United States of America among 1015 respondents, showed that those with low household income express a high level of anxiety [44]. A SARS previous study further elucidates that infectious disease outbreak affect the economic and social patterns of people's life which also have detrimental impacts on their psychological health [46]. Hence, it is necessary to make sure that mental health intervention concentrates on the part of the population facing severe social household economic burdens. Regarding residential area association with anxiety levels, COVID-19 survey among the general population in Spain observed that people living in rural areas had high anxiety compared to those living in urban settlements, this is consistent with this present study [47]. In contrast, another study in same region on sociodemographic characteristics of respondents confined due to COVID-19, reported that both those that settle in urban and rural areas, exercise the same level of anxiety [48]. A disconnection from the government equipping health institutions in rural settlements have led to worse health awareness against the pandemic [49]. Therefore, healthcare providers and NGOs should include and prioritize rural settlement in their actions during pandemics. Also, health promotion of mental health during outbreaks of infectious diseases to help reduce the perceived threat, social and household economic effects, and to help the most vulnerable people is essential.

**Analyses of the relationship between knowledge, attitude, and anxiety levels using logistic regression:** this study showed higher odds for moderate to high anxiety among participants with fair/poor knowledge than those with good knowledge (cOR=2.32, 95% CI: 1.38-3.91). This means that respondents with good knowledge about the COVID-19 pandemic have low anxiety levels. This was in line with a report from France during the first and second waves of the pandemic, which said that people with a good understanding of how the disease spreads, who follow containment measures, and who do what the health authorities say to stop the virus seem to have less anxiety [50]. Researchers in Iran at the end of the pandemic found that those who knew the most about it had the least anxiety [51], and those with moderate and low knowledge are likely to have high anxiety [52]. In contrast, many other surveys have indicated a negative association of anxiety with knowledge [18,53,54]. However, this study also showed that having a high level of education makes people more knowledgeable and aware of the ongoing health crises, which makes them less worried in times of pandemics. Good knowledge give rise to positive attitudes which reduces anxiety among Chinese studying and living abroad [55]and a report from Bangladesh among college student also highlighted that anxiety levels of students with positive attitudes was low [27]. Although our study, shows no significant relationship between attitudes and anxiety levels after the lift of the pandemic lockdown, it is possible that, the population had already acquired knowledge about the pandemic and it is now part of them to routinely practice preventive measure against the pandemic. Dissimilar results were observed among an adult population in Turkey which showed strong relationships between the perception and attitude towards the coronavirus pandemic scale and anxiety [56]. In United Arab Emirates, there was a positive link and a statistically significant difference between the study group's attitude level and their anxiety and psychological preparation which also contradicts the results of the present study [57]. The findings of our study provide unique information that will be useful for healthcare providers and policymakers in areas of knowledge, attitudes and anxiety among general population and particularly young people during the pandemic, furthermore, findings should only be considered exploratory and as a basis for future studies using GAD-7, the English version, as the primary measurement instrument for anxiety.

**Limitation and strength of the study:** there are a few things to keep in mind when trying to figure out what the study's results mean. Because this was a cross-sectional study, it was hard to figure out what caused the link between knowledge and anxiety. The validity of the findings was affected by questions about the measurement of knowledge, attitude, and stress related to the COVID-19 pandemic after the lockdown. It's important to note that this study was only done on young people who had access to the internet and had an average socioeconomic status. This means that the results cannot be used outside of this specific context. The main strength of our study is that it gives essential information to health authorities, policymakers, and organizations about COVID-19 related anxiety in a vulnerable group. This information has the potential to help with efforts to control and prevent the COVID-19 Pandemic among young people in the South-Eastern part of Nigeria and could also be used in the circumstance of a future health crisis.

## Conclusion

This cross-sectional survey showed that people in five South-Eastern Nigerian States knew a lot, had a good attitude and were not too worried after the lift of COVID-19 pandemic lockdown. Anxiety was linked to a person's level of education, household income, and where they lived. We also found a link between COVID-19 knowledge and anxiety level in the study population. This suggests that people with more education, a higher income, and who live in cities have a better understanding of COVID-19 and less anxiety. There was no link between how someone felt and how anxious they were. The results of our study give unique information that will be useful for health care providers and policymakers about what people know, how they feel about it, and how worried they are, especially young people, during pandemics. During outbreaks of infectious diseases, promoting mental health can help reduce the perceived threat, social and household economic effects, and help the most vulnerable people.

### What is known about this topic

- According to experts, understanding COVID-19 transmission and symptoms will aid in pandemic control;
- Previous research has found moderate to high levels of fear during the pandemic lockdown, particularly among young people;
- Studies have also revealed that academic exposure helps position the youths to increase access to accurate information.

### What this study adds

- After the lockdown was lifted, most young people in Nigeria's South-Eastern state had a good understanding of how the COVID-19 pandemic spreads and what its symptoms are, as well as a positive attitude;
- This study shows that young adults in South-Eastern Nigeria who know a lot about COVID-19 disease have less anxiety after the lockdown; there was no link between the population's attitude and level of their anxiety;
- Lastly, the level of anxiety seems to be linked to how much schooling someone has, how much money their family makes, and where they live; it means that people who have higher education qualification, make a good living, and live in a city have less anxiety than those who don't.
